# Visual Evaluation of Plethysmographic Waveforms: Introducing the Simple Systolic Ratio as an Indicator of Fluid Responsiveness

**DOI:** 10.4274/TJAR.2024.231452

**Published:** 2024-02-28

**Authors:** Muhammet Selman Söğüt, Kamil Darçın, Muhammet Ahmet Karakaya, Mete Manici, Yavuz Gürkan

**Affiliations:** 1 Koç University Hospital, Department of Anaesthesiology and Reanimation, İstanbul, Turkey; 2 Acıbadem Ataşehir Hospital, Clinic of Anaesthesiology and Reanimation, İstanbul, Turkey

**Keywords:** Cancellation, elective surgeries, perioperative care, preoperative assessment, surgery scheduling

## Abstract

**Objective:**

For patient safety, maintaining hemodynamic stability during surgical procedures is critical. Dynamic indices [such as systolic pressure variation (SPV) and pulse pressure variation (PPV)], have recently seen an increase in use. Given the risks associated with such invasive techniques, there is growing interest in non-invasive monitoring methods-and plethysmographic waveform analysis. However, many such non-invasive methods involve intricate calculations or brand-specific monitors. This study introduces the simple systolic ratio (SSR), derived from pulse oximetry tracings, as a non-invasive method to assess fluid responsiveness.

**Methods:**

This prospective observational study included 25 adult patients whose SPV, PPV, and SSR values were collected at 30-min intervals during open abdominal surgery. The SSR was defined as the ratio of the tallest waveform to the shortest waveform within pulse tracings. The correlations among SSR, SPV, and PPV were analyzed. Additionally, anaesthesia specialists visually assessed pulse oximetry tracings to determine fluid responsiveness using the SSR method.

**Results:**

Strong correlations were observed between SSR and both SPV (r = 0.715, *P* < 0.001) and PPV (r = 0.702, *P* < 0.001). Receiver operator curve analysis identified optimal SSR thresholds for predicting fluid responsiveness at 1.47 for SPV and 1.50 for PPV. A survey of anaesthesia specialists using the SSR method to visually assess fluid responsiveness produced an accuracy rate of 83%.

**Conclusion:**

Based on the strong correlations it exhibits with traditional markers, SSR has great potential as a clinical tool, especially in resource-limited settings. However, further research is needed to establish its role, especially as it pertains to its universal applicability across monitoring devices.

**Main Points**

• This prospective observational study introduces the simple systolic ratio (SSR) from pulse oximeter tracings as a non-invasive method to assess fluid responsiveness during surgery.

• SSR is defined as the ratio of the tallest waveform to the shortest waveform within the pulse tracings.

• Twenty five adult patients undergoing open abdominal surgery were observed, comparing SSR with the traditional markers systolic pressure variation (SPV) and pulse pressure variation (PPV).

• Strong correlations were found between SSR and both SPV (r = 0.715) and PPV (r = 0.702).

• Receiver operator curve analysis identified the optimal SSR threshold for predicting fluid responsiveness as 1.50.

• A survey with anaesthesia specialists demonstrated an accuracy rate of 83% in visually assessing fluid responsiveness using the SSR method.

## Introduction

Hemodynamic stability during surgical procedures is vital for patient safety. Maintaining optimal intravascular volume and ensuring proper fluid therapy are especially crucial during open abdominal surgery. In recent years, dynamic indices, such as systolic pressure variation (SPV) and pulse pressure variation (PPV), have become essential tools for assessing the fluid responsiveness of intubated patients.^1,2^ Although these parameters provide valuable insights, their invasive nature may not be suitable or feasible for every patient or monitoring apparatus.

Given the challenges and potential complications associated with invasive methods, there is growing interest in non-invasive monitoring techniques. In particular, plethysmographic waveform analysis is a promising method for assessing fluid status in patients.^3,4,5^ Although numerous indices derived from plethysmographic waveforms can forecast fluid responsiveness, their practical application is often hindered by the need for intricate computations, specialized algorithms, or brand-specific monitors, which curtail their universal applicability and adoption across diverse clinical settings.

Several of the aforementioned indices employ formulas based on the amplitudes of the plethysmographic waveforms. A closer inspection of these formulas reveals an underlying simplicity: they center predominantly around contrasting the amplitude of the tallest waveform with that of the shortest. Despite being cloaked in complex terminology and mathematical representations, the essence of these methods can be distilled to a basic ratio of the tallest wave to the shortest wave in the waveform.

A widely recognized index derived from plethysmography waveform analysis is the pulse oximetric plethysmographic (POP) waveform amplitude index,^6^ which is described by the following formula:

$\mathrm{POP}\;=\frac{{\mathrm{POP}}_{\max}\;-\;{\mathrm{POP}}_{\min}}{\frac{{\mathrm{POP}}_{\max}\;+\;{\mathrm{POP}}_{\min}}{2}}$

In this formula, POP_max_ and POP_min_ represent the heights of the tallest and shortest waves , respectively. By introducing a variable, R (where R is the ratio of POP_max_ to POP_min_ (POP_max_ ⁄ POP_min_), and rearranging the formula accordingly, it becomes evident that the POP formula essentially translates to R accompanied by some constants (POP=2R – 2⁄ R + 1), highlighting that the core of the formula lies not in the absolute values of POP_max_ and POP_min_ but in their comparative relationship. Consequently, simply measuring the ratio R may suffice in making predictions similar to those made by the POP index.

Furthermore, direct visual interpretation of the plethysmographic waveform to estimate the ratio R, which is referred to as the simple systolic ratio (SSR) in this study, without relying on specialized tools or intricate calculations, may offer insights comparable to advanced methods. SSR is closely linked to the POP index because it is derived from the same formula and can reflect dynamic changes during the respiratory cycle as the POP index does.^6^ By simplifying the formula used in POP measurements, SSR was developed to provide a straightforward yet effective approach, free from the constraints of brand-specific equipment and complex calculations. Such a method is particularly valuable in resource-limited settings, including developing countries and remote areas, as it provides clinicians with a practical tool for monitoring hemodynamic changes during surgeries.

The central hypothesis of this study is that SSR is correlated with the established fluid responsiveness parameters SPV and PPV and can therefore be used as a non-invasive monitoring technique to assess fluid responsiveness. This investigation validates SSR against these conventional metrics and assess its practical utility in clinical settings. A key focus is to determine the feasibility of anaesthesia professionals to visually discern fluid responsiveness by interpreting SSR.

## Methods

Ethical approval for this prospective observational study was granted by the Koç University Hospital Clinical Research Ethics Committee (approval no: 2020.439.IRB1.162, date: 26.11.2020).

A cohort of 25 adult patients scheduled for open abdominal surgery was enrolled and participated in this study. The primary inclusion criterion for the study was imminent open abdominal surgery, with patients included only when the attending anaesthesiologist deemed invasive arterial pressure monitoring medically necessary. Informed consent was obtained from all study participants.

To eliminate potential confounders, the following exclusion criteria were established:

1. Refusal to participate. Patients from whom informed consent was not obtained were excluded from the study.

2. Presence of advanced cardiac disease. Patients with a history of (or current) severe cardiac disease that could affect hemodynamic parameters or their interpretation were excluded from the study. Advanced cardiac disease was defined as anyone or both of the following parameters:

• An ejection fraction <35%, as assessed by echocardiography;

• Class III/IV heart failure, as defined by the New York Heart Association.

3. Presence of rhythm abnormalities. Because cardiac rhythm disturbances can affect the accuracy of plethysmographic waveforms and other derived parameters, patients with arrhythmias or any significant rhythm abnormalities were excluded from the study.

4. Presence of pulmonary hypertension. Because pulmonary hypertension can introduce changes in hemodynamic response and could confound the interpretation of plethysmographic waveforms, patients with a mean pulmonary arterial pressure >25 mmHg at rest, as measured by right heart catheterization or echocardiography, were excluded.

5. Use of vasoactive medications. Patients on medications that significantly influence vascular tone and hemodynamics were not considered suitable for this study.

**Image 1:**
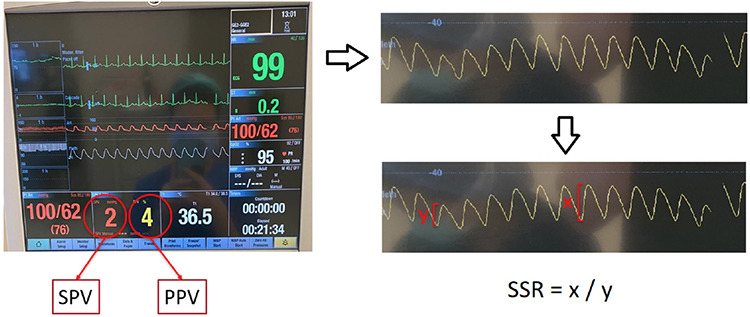
Process of data extraction from screen images. SSR, simple systolic ratio; SPV, systolic pressure variation; PPV, pulse pressure variation.

Screen captures from anaesthesia monitors (Datex-Ohmeda CARESCAPE B850; GE Healthcare; Chicago, IL) were taken at 30-min intervals while patients underwent surgery. SPV and PPV values were extracted from this screen captures by a researcher who then isolated the plethysmography waveform from each capture to create new image files. To maintain objectivity and ensure blindness, a researcher who was uninformed about the previously extracted data was subsequently tasked with employing pixel counts to compute SSR values from the new image files. The SSR was defined as the ratio of the tallest waveform to the shortest waveform within pulse tracings (Image 1).

### Statistical Analysis

The normal distribution of each variable was evaluated using the Kolmogorov-Smirnov test. Variables adhering to a normal distribution are summarized as means and standard deviations. Variables that demonstrated a non-normal distribution are summarized as median and range.

The correlations between SSR, SPV, and PPV values were assessed using Spearman or Pearson correlation analysis based on the normal or non-normal distribution of the variables.

To determine diagnostic accuracy and the threshold value of SSR in predicting fluid responsiveness, two receiver operator curve (ROC) analyses were performed: one each for SPV and PPV as the determinant of the state variable. In the first ROC analysis, an SPV value of 10 was used as the threshold.^7^ Cases where the SPV was equal to or exceeded 10 were categorized as “1”, and cases where the SPV was below 10 were categorized as “0”. This state variable and the SSR values were used to create an ROC curve, which was used to analyze the SSR’s ability to diagnose these cases accurately. Similarly, in the second ROC analysis, a PPV value of 15 served as the threshold.^8^ Cases with PPV values of 15 or higher were categorized as “1”, and a ROC curve was created using this state variable and SSR values. The optimal threshold for SSR was determined by calculating the value that maximized the equation (sensitivity + specificity)/2 for each of the two ROC analyses separately.

All data analyses were performed using SPSS v. 24.0 statistical software. Results were considered statistically significant at *P* < 0.050.

In a separate segment of the study, 20 pulse oximetry tracings with SSR values ranging from 1.09 to 2.12 were presented to anaesthesia specialists, who were asked to visually assess the waveforms and decide whether the SSR of the waveform was greater than the threshold established in the ROC analysis. The accuracy of the answers provided in the survey was then compared with the preestablished SSR values. The purpose of this survey was to evaluate the ability of anaesthesia specialists to visually estimate fluid responsiveness using the SSR method.

A sample size analysis was conducted before patient recruitment. The analysis was based on the following parameters: α (two-tailed) = 0.05, β = 0.10, and an expected correlation coefficient (r) of 0.60. The selection of a correlation coefficient of 0.60 was based on the prediction of a moderate to strong correlation between the SSR and both SPV and PPV, owing to the underlying mathematical equivalence of POP and SSR and previously established correlations between POP and SPV. In Addison et al.,^9^ various signal processing algorithms applied to POP yielded correlation coefficients ranging from 0.35 to 0.85 with SPV. A coefficient of 0.60 was chosen to represent a moderate value within this range, aligning with the expected correlation strength for the purposes of this study. Based on these parameters, the sample size analysis indicated that a sample size of 25 would be sufficient to reliably detect and validate the proposed correlations.

## Results

The study consisted of 13 male and 12 female patients with an average age of 54 years [range: 38-70 y, standard deviation (SD): 10.6]. The mean body mass index of the participants was 26.4 kg m^2-1^ (range: 20.5-32.8 kg m^2-1^, SD: 3.9). The primary indications for surgery among study participants included gastrointestinal tumors (n = 12), hernias (n = 7), and other abdominal pathologies (n = 6). All patients had an American Society of Anesthesiologists physical status score of II or III.

A total of 117 screen captures were obtained during the open abdominal surgeries of the study participants. For each screen capture, the SSR, SPV, and PPV values were successfully extracted without any data loss.

The Kolmogorov-Smirnov test revealed that the SSR, SPV, and PPV data did not follow a normal distribution (Table 2). Spearman correlation analysis revealed a strong correlation between SSR and SPV, with a correlation coefficient (r) of 0.715. This result was statistically significant (*P* < 0.001), suggesting a strong positive relationship between the two parameters (Table 2). Similarly, a strong correlation was observed between SSR and PPV, with a correlation coefficient of 0.702. This result was also statistically significant (*P* < 0.001) (Table 2).

**Image 2:**
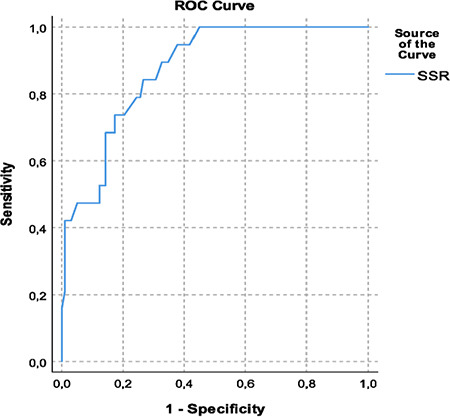
Systolic pressure variation based receiver operating characteristic curve analysis of simple systolic ratio. SSR, simple systolic ratio; ROC, receiver operating characteristic.

The first ROC analysis of SSR values, conducted using SPV values as the determinant of the state variable, yielded an area under the curve of 0.873 with a standard error of 0.037 (*P* > 0.001) and an optimal SSR threshold of 1.47 with a Youden’s J index of 0.577 (Image 2). In predicting elevated SPV values, SSR exhibited a sensitivity of 83% and specificity of 74% at this threshold.

**Image 3:**
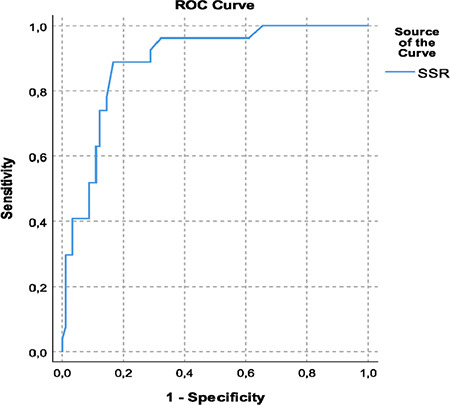
Pulse pressure variation based receiver operator curve analysis of simple systolic ratio. SSR, simple systolic ratio; ROC, receiver operating characteristic.

The second ROC analysis of SSR values, conducted using PPV values as the determinant of the state variable, yielded an area under the curve of 0.890 with a standard error of 0.033 (*P* > 0.001) and an optimal SSR threshold of 1.50 with a Youden’s J index of 0.722 (Image 3). Applying this threshold, SSR registered a sensitivity of 89% and specificity of 83%.

Given the proximity of the optimal SSR thresholds identified for SPV and PPV, a pragmatic approach for the survey was adopted, in which the SSR threshold was unified at 1.50. This decision was motivated by the desire to facilitate the task set out for the survey participants. Asking participants to determine whether a ratio surpassed a specific value, such as 1.47, would have been logistically challenging and potentially imprecise; therefore, the rounded-off value of 1.50 was chosen as the uniform SSR threshold for the survey, ensuring a more straightforward and feasible query for the participating anaesthesia experts.

During the survey of anaesthesia experts, each of the 28 experts was presented with a set of 20 plethysmographic waveforms, the variations of which represented SSR values ranging from 1.09 to 2.12. The participants’ primary task was to visually inspect each waveform and determine whether the ratio of the tallest waveform to the shortest surpassed the 1.5 SSR threshold established by the earlier ROC analysis.

**Image 4:**
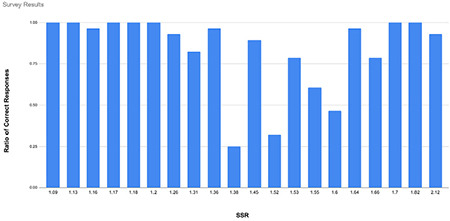
Results of the survey of anesthesia experts. SSR, simple systolic ratio.

Of the 560 responses gathered (28 × 20), 467 were correct, representing an overall accuracy rate of 83%. However, it was observed that for waveforms with an SSR value between 1.38 and 1.6, the accuracy rate dropped to 55%. Conversely, for waveforms that fell outside this range, the accuracy rate climbed to 95% (Image 4).

## Discussion

The primary aim of this study was to explore the feasibility and accuracy of SSR as a non-invasive tool for determining fluid responsiveness in patients undergoing open abdominal surgery. The studys findings provide substantial evidence supporting the efficacy of SSR in this role.

A significant correlation between SSR and both SPV and PPV emerged during the analysis, suggesting that SSR could serve as an effective surrogate marker for these established indicators. Given that SPV and PPV have long been upheld as reliable markers of fluid responsiveness, the strong association between SSR and these indices further underscores the potential of SSR as a valuable clinical tool.

Pulse oximetry sensors and monitors use digital filters to refine and clarify plethysmographic waveforms, ensuring that clinicians receive readable and consistent signal.^10,11^ However, the exact specifications and characteristics of these filters can vary significantly across different sensors and monitor brands. Consequently, the SSR threshold determined in this study may not be universally applicable across devices. Clinicians should exercise caution before adopting the 1.5 threshold globally, as it could lead to misinterpretations and potential clinical errors. Rather than relying on specific values at isolated time points, observing SSR trends over time may offer a more reliable and holistic insight into patients’ fluid responsiveness. To better define device-specific thresholds and refine the use of SSR, additional research involving various monitors and sensors is required to capture the broad spectrum of technology currently in clinical use.

This study illuminated the potential of straightforward visual assessment of plethysmographic waveforms as a powerful indicator of fluid responsiveness; however, it also revealed challenges, particularly in recognizing SSR values near the chosen threshold. While overall accuracy registered at 83%, this dropped to 55%, which is close to the established threshold. Nevertheless, this limitation may be easily mitigated by temporarily freezing the monitor’s display when in doubt and enlisting the help of a simple ruler to measure the tallest and shortest waves. This approach can increase the reliability of the SSR method, making it a valuable tool in resource-constrained environments where sophisticated hemodynamic monitoring equipment may be scarce.

### Study Limitations

This study was based on a relatively small sample size of 25 patients undergoing similar surgeries and thus may not be representative of the broader population.

This study used SPV and PPV as reference indices; however, no single index is perfect, and another index may offer a different perspective on the effectiveness of SSR. This study used a specific pulse oximeter and monitor. Because different monitors and sensors may have unique digital filters that affect plethysmographic waveforms, the study’s findings may not be directly applicable to devices from other manufacturers.

## Conclusion

This study shows the potential of SSR as an efficient, non-invasive tool for assessing fluid responsiveness through visual analysis of plethysmographic waveforms. Further research and validation across diverse clinical contexts will be instrumental in establishing the role of SSR in routine clinical practice.

## Figures and Tables

**Table 1:**
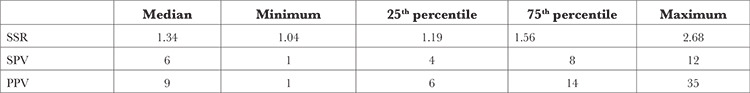
Summaries of SSR, SPV and PPV Measurements

**Table 2:**
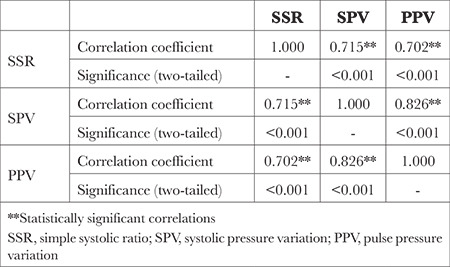
Spearman’s Correlation Analysis of Simple Systolic Ratio, Systolic Pressure Variation, and Pulse Pressure Variation
